# GEDAE-LaB: A Free Software to Calculate the Energy System Contributions during Exercise

**DOI:** 10.1371/journal.pone.0145733

**Published:** 2016-01-04

**Authors:** Rômulo Bertuzzi, Jorge Melegati, Salomão Bueno, Thaysa Ghiarone, Leonardo A. Pasqua, Arthur Fernandes Gáspari, Adriano E. Lima-Silva, Alfredo Goldman

**Affiliations:** 1 Endurance Performance Research Group (GEDAE-USP), School of Physical Education and Sport, University of São Paulo, São Paulo, Brazil; 2 Sports Science Research Group, Department of Physical Education and Sports Science (CAV), Federal University of Pernambuco, Pernambuco, Brazil; 3 Systems and Software Group, Department of Computer Science, University of São Paulo, São Paulo, Brazil; East Tennessee State University, UNITED STATES

## Abstract

**Purpose:**

The aim of the current study is to describe the functionality of free software developed for energy system contributions and energy expenditure calculation during exercise, namely GEDAE-LaB.

**Methods:**

Eleven participants performed the following tests: 1) a maximal cycling incremental test to measure the ventilatory threshold and maximal oxygen uptake ($\dot{\text{V}}$O_2_max); 2) a cycling workload constant test at moderate domain (90% ventilatory threshold); 3) a cycling workload constant test at severe domain (110% $\dot{\text{V}}$O_2_max). Oxygen uptake and plasma lactate were measured during the tests. The contributions of the aerobic (A_MET_), anaerobic lactic (LA_MET_), and anaerobic alactic (AL_MET_) systems were calculated based on the oxygen uptake during exercise, the oxygen energy equivalents provided by lactate accumulation, and the fast component of excess post-exercise oxygen consumption, respectively. In order to assess the intra-investigator variation, four different investigators performed the analyses independently using GEDAE-LaB. A direct comparison with commercial software was also provided.

**Results:**

All subjects completed 10 min of exercise at moderate domain, while the time to exhaustion at severe domain was 144 ± 65 s. The A_MET_, LA_MET_, and AL_MET_ contributions during moderate domain were about 93, 2, and 5%, respectively. The A_MET_, LA_MET_, and AL_MET_ contributions during severe domain were about 66, 21, and 13%, respectively. No statistical differences were found between the energy system contributions and energy expenditure obtained by GEDAE-LaB and commercial software for both moderate and severe domains (*P* > 0.05). The ICC revealed that these estimates were highly reliable among the four investigators for both moderate and severe domains (all ICC ≥ 0.94).

**Conclusion:**

These findings suggest that GEDAE-LaB is a free software easily comprehended by users minimally familiarized with adopted procedures for calculations of energetic profile using oxygen uptake and lactate accumulation during exercise. By providing availability of the software and its source code we hope to facilitate future related research.

## Introduction

The establishment of the amount of energy expenditure during exercise has been considered of key importance for strategy development aiming at both health [1] and athletic performance [2] improvement. Over the past century, many methods have emerged in literature in order to assess energetic profiles during dynamic exercise [3,4]. It has been widely accepted that whole-body oxygen uptake ($\dot{\text{V}}{\text{O}}_{2}$) can be used to represent aerobic system contribution (A_MET_) [5]. On the other hand, there is no universal method accepted as gold standard to estimate anaerobic system contribution [5,6]. It has been proposed that measurements of muscle metabolites using biopsy could provide relevant information regarding alactic (AL_MET_) and lactic (LA_MET_) anaerobic systems [7]. Aside from being an invasive procedure, the small sample size taken in the biopsy for the determination of maximum amount of ATP that can be resynthesized by anaerobic system might result in an inaccurate estimative [6]. Thus, alternative methods using whole-body physiological variables have been proposed in order to estimate the anaerobic system contribution (AL_MET_ and LA_MET_) during dynamic exercises.

Among the main methods using whole-body physiological variables to estimate AL_MET_ and LA_MET_, the measurement of post-exercise $\dot{\text{V}}{\text{O}}_{2}$ and blood lactate accumulation during exercise has been probably the most widely used [2,8,9]. In this model, AL_MET_ is assessed by the determination of the fast component of excess post-exercise oxygen consumption [10,11], while LA_MET_ is assessed by calculating the lactate accumulation O_2_ equivalent [12], respectively. Thus, the sum of AL_MET_ and LA_MET_ provide the total anaerobic contribution. Previous studies have demonstrated that this non-invasive method is able to properly assess the energy amount supplied by the anaerobic system when compared with other traditional methods such as maximal accumulated oxygen deficit [8] and Wingate test [9]. Furthermore, this method also has the additional advantage of enabling the establishment of energetic profile using portable metabolic system in a large variety of non-laboratory-based activities, such as weight training [2], rock climbing [13], ballet [14], and karate [15]. The convenience of this method is especially important because the increase of knowledge with regard to energy systems contributions during real life situations might provide a greater effectiveness of training strategies to improve both health and athletic performance.

Although this method provides many methodological advantages, it requires the use of some mathematical functions that may be unusual for some exercise physiologists. For example, in order to determine the AL_MET_ during exercise is necessary to fit the post-exercise $\dot{\text{V}}{\text{O}}_{2}$ kinetics using a bi- or a mono-exponential model [8,9,16]. In addition, the area over the time used to represent the A_MET_ has been determined using a trapezoid rule [14,17,18]. In addition, although the A_MET_ and LA_MET_ can be determined by a single interactive spreadsheet computer program (e.g. Excel), the determination of the AL_MET_ is substantially more complex because it is necessary to choose appropriate exponential equations and insert first-estimated values for each parameter for those are not automatically provided. Given that computer capabilities have been improved over the last decades, an array of emerging software packages have assisted in these data analyses. However, because available software packages are not free and were not designed especially for energy expenditure determination, they still might be considered costly, time-consuming, expertise-requiring and error-prone in their use. Unfortunately, the lack of direct available software may discourage physiologists from determining the metabolic profile during exercise.

Thus, the aim of the present study was to develop open-source software with an easy-to-use interface designed to calculate the energy system contributions and energy expenditure during exercise, using measurements of $\dot{\text{V}}{\text{O}}_{2}$ and blood lactate accumulation. In addition, we also described GEDAE-LaB’s development, system requirements, advantages, limitations, and future directions for this software. A direct comparison with a conventional commercial software and measurement of reliability of the GEDAE-LaB across different exercise physiologists were also provided.

## Materials and Methods

### Participants

Eleven male students of physical education (age 25.1 ± 6.0years; height 175.3± 8.6 cm; and body weight 74.1 ± 3.0 kg), accustomed to exhaustive exercise, volunteered to participate in this study. The participants had been training 3–4 times per week in recreational sports (jogging, soccer, and cycling) for at least one year. The participants were free of neuromuscular and cardiovascular dysfunctions and were not taking any medications at the time of data collection. They received a verbal explanation of the possible benefits, risks, and discomfort associated with the study and signed a written informed consent before participating in the study. The study was conducted in accordance with the Helsinki Declaration of 1975, and was approved by the Ethics Committee for Human Studies of the Federal University of Pernambuco (process number: 039412/2013).

### Experimental design

The participants visited the laboratory on three separate occasions. They were submitted to the following experimental sessions: 1) a maximal cycling incremental test to measure the ventilatory threshold and maximal oxygen uptake ($\dot{\text{V}}$O_2_max); 2) a cycling workload-constant test at moderate domain (90% ventilatory threshold); 3) a cycling workload-constant test at severe domain (110% $\dot{\text{V}}$O_2_max). These workload-constant tests were carried out in a counterbalanced order. Each test was performed in two different occasions, at least 48 hours apart, in the same time of the day to avoid any circadian interference [19]. The oxygen uptake and plasma lactate accumulation were measured during workload-constant tests to determinate energy expenditure profile using GEDAE-LaB. The participants were instructed to refrain from exhaustive exercises and caffeine during the 24h preceding the testing sessions, and to avoid food intake about 2 h before the tests. Subjects were also instructed to maintain the same diet habits throughout the study. All tests were performed in different days in a controlled laboratory environment (20.6 ± 1.8°C and 54.2 ± 3.7% relative humidity). In order to assess the intra-investigator variation, four different investigators performed the analysis using the GEDAE-LaB. Only one investigator was an expert in the procedures adopted for the calculations of energy expenditure, while others were not familiarized with these procedures. The investigators that were not familiarized with the calculations were instructed to perform each phase of the calculations using only the information contained in the GEDAE-LaB. The results were blinded among the investigators during analysis.

### Maximal cycling incremental test

The maximal incremental exercise test was carried out on an electromagnetically braked cycle ergometer (Ergo Fit 167, Pirmansens, Germany). After a 5 min warm-up at 25 W, participants exercised at a pedal frequency of 60 rpm with initial intensity of 50 W and power output increments of 25 W∙min^-1^ until exhaustion [10]. In order to attain maximum values, participants received strong verbal encouragement to continue as long as possible. The maximal heart rate (HRmax) was defined as the highest value obtained at the end of the test. The maximal oxygen uptake ($\dot{\text{V}}$O_2_max) was considered as the average of the oxygen uptake measured during the last 30 s of the test [20]. Maximal power output was established as the power output that elicited $\dot{\text{V}}$O_2_max. As previously described [21], the first ventilatory threshold was visually determined by two independent investigators using the following criteria: a) the inflection point of VCO_2_ with respect to $\dot{\text{V}}{\text{O}}_{2}$, b) the point of a nonlinear increase in $\dot{\text{V}}\text{E}/\dot{\text{V}}{\text{O}}_{2}$, c) the increase in PETO_2_ while PETCO_2_ remained stable. When investigators were not in agreement, a third independent investigator was consulted.

### Cycling workload-constant tests

The same cycle ergometer and gas analyzer used in the maximal incremental test were used during the workload-constant tests. Before tests, the participants rested quietly on the cycle ergometer for 5 min to measure resting $\dot{\text{V}}$O_2_values ($\dot{\text{V}}$O_2baseline_). Subsequently, they exercised during 10 min or until the exhaustion at two different workloads (moderate or severe exercise domains). Exhaustion was defined as the incapacity to maintain a minimum pedal cadence of 60 rpm. The recovery time used to obtain the fast component of the excess post-exercise oxygen consumption was 10 min [8].

### Physiological measurements

Gas exchanges were measured breath-by-breath using a gas analyzer calibrated according to manufacturer recommendations (Cortex Metalyzer 3B, Cortex Biophysik, Leipzig, Germany). Heart rate was measured using a heart rate transmitter (Polar Electro Oy, Kempele, Finland) connected to the gas analyzer. During the cycling workload-constant test, the blood samples (25 μl) were collected from the ear lobe during rest, immediately, and three and five minutes after exercise to determine the peak plasma lactate concentration ([La^-^]_peak_), using enzymatic calorimetric reactions in a spectrophotometer, as previously described [20].

### Development of GEDAE-LaB

In this first version, GEDAE-LaB allows the determination of the contribution of each energy system using oxygen uptake measurement during and post exercise and blood or plasma lactate accumulation. It is a free software, intentioned to facilitate analysis of energetic profile during exercise. The GEDAE-LaB is available online and does not require any download, registration or login. It can be accessed at http://www.gedaelab.org. The detailed statistics about software`s traffic and traffic sources will be generated in the future by Google Analytics.

This software was developed using Extreme Programming software development methodology [22] that focuses primarily on customer satisfaction. This goal is achieved through several practices, including short development cycles and client presence; that is, the second and third authors were always present during development team working sessions. Since the doubts of programmers could be rapidly answered, the correct features were rightly implemented, avoiding unnecessary rework. Still based on Extreme Programming, the development team also created unit and acceptance tests that were run automatically after every change in the code was pushed to the repository, which enabled quicker responses to programming errors. The trustworthiness created on tests allowed for the use of continuous integration, that is, after every code change committed, a new software version was put live on the server. The software development team worked in pairs that changed every session, so any programmer would know most of the software codebase and would be able to change anything–what is called collective ownership. The software was implemented as a website which allows anyone with Internet access to use it without installing anything. The only requirement is a JavaScript-enabled browser, such as Internet Explorer, Mozilla Firefox, Google Chrome and Apple Safari. The energy expenditure calculation is made on server side, which demands even less from client hardware. The server side application was developed using Java programming language with the VRaptor MVC (http://www.vraptor.org/) framework. Tests were created using Junit (http://junit.org/) (unit tests) and Selenium (http://www.seleniumhq.org/) (acceptance tests) and continuous integration was implemented through JenkinsCI (https://jenkins-ci.org/). All dependencies were handled using Maven (https://maven.apache.org/). The application runs on a Tomcat webserver running a Linux operating system. The project code is available on GitHub (https://github.com/physusp/physusp).

It is very important to observe that, aiming to serve a wide number of users, GEDAE-LaB was developed using English decimal separator. Thus, physiological data must be inputted using comma and point as a fractional part for thousand and decimal, respectively. The duration time of the measurement of the $\dot{\text{V}}$O_2_ during exercise and recovery must be expressed using seconds (s) or using the format hh:mm:ss. In addition, $\dot{\text{V}}$O_2_ and lactate concentrations must be expressed in ml∙min^-1^and mmol∙l^-1^ (or mM), respectively. These physiological data can be copied and pasted from their respective spreadsheets in the software.

### Calculations of the energy expenditure

The first template of GEDAE-LaB contains brief information about the software, followed by a second template where the users choose which energy system they wish to calculate. The A_MET_, AL_MET_, and LA_MET_ can be calculated together or individually. The mathematical functions used in GEDAE-LaB to estimate energetic profile during dynamic exercise were in accordance with previous studies [13,14,15]. The trapezoidal method is used to calculate the oxygen uptake area over time during exercise and thus to estimate A_MET_.

To calculate A_MET_, the $\dot{\text{V}}$O_2_ baseline can be ignored, measured or assumed as a fixed value (i.e. 3.5 ml∙kg^-1^∙min^-1^) [23]. In the analyses presented in this study, we measured $\dot{\text{V}}$O_2_ rest, which was automatically subtracted from oxygen uptake area by the software to obtain A_MET_. The AL_MET_ is considered the fast component of excess post-exercise oxygen consumption. The users have to choose one of these models to calculate AL_MET_. In the present study, we used a mono-exponential model to fit oxygen uptake data after moderate exercise domain and bi-exponential model after severe exercise domain. Previous findings have suggested that the $\dot{\text{V}}$O_2_ responses after exercise performed at moderate domain is better described by mono-exponential model, while the $\dot{\text{V}}$O_2_ responses after exercise performed at severe domain is better described by bi-exponential [24]. In order to analyze a possible impact of the time of recovery on calculation of the AL_MET_, the fast component of excess post-exercise oxygen consumption was determined using the $\dot{\text{V}}$O_2_ data during 6, 8, and 10 min of recovery. To improve fitting the oxygen uptake data, some optimization rules were used. The $\dot{\text{V}}$O_2baseline_ had to be fixed, based on previous phase calculation, and also the fit converges. In both cases, a least squares problem is solved using the Levenberg-Marquardt algorithm. For the bi-exponential model, the initial guess values were based on a mono-exponential fit. The Eq 3 is automatically applied to obtain the contribution of the AL_MET_. The A_MET_ and AL_MET_ were also calculated using commercially available software (Origin 6.0, Microcal, Massachusetts, USA), while the LA_MET_ was calculated using an interactive spreadsheet computer program (Microsoft Excel for Mac 2011, Version 10.0.0, California, USA).

The LA_MET_ was estimated using the oxygen equivalent from blood lactate accumulation. It was determined as the difference between [La^-^]_peak_ and resting blood lactate concentration [La^-^]_rest_, expressed as a delta value ([La^-^]_net_). A value of 1 mmol∙l^-1^ [La^-^]_net_ is considered to be equivalent to 3 ml O_2_∙kg^-1^ body mass and is used to obtain LA_MET_ contribution. The user has to include both rest [La^-^]_rest_ and [La^-^]_peak,_ and body mass, so [La^-^]_net_ and LA_MET_ will be automatically calculated. The energy expenditure and energy systems were expressed in liters of oxygen, kilojoules and kilocalories, assuming caloric equivalents of 20.9 kJ∙LO_2_^-1^ and 5 Kcal∙LO_2_^-1^ for kilojoules and kilocalories, respectively. Total energy expenditure was calculated as the sum of the three energy systems (A_MET_ + AL_MET_ + LA_MET_). In addition, the contributions of the three energy systems were also expressed as total energy expenditure percentage.

$$\dot{\text{V}}{\text{O}}_{2(\text{t})}=\;\dot{\text{V}}{\text{O}}_{2\text{baseline}}+\;{\text{A}}_{1}[e_{1}^{-(t-td)/t_{1}}]$$ (1)

$$\dot{\text{V}}{\text{O}}_{2(\text{t})}=\;\dot{\text{V}}{\text{O}}_{2\text{baseline}}+\;{\text{A}}_{1}[{\text{e}}^{-(\text{t}-\text{td})/t_{1}}]\;+\;{\text{A}}_{2}[e^{-(t-td)/t_{2}}]$$ (2)

$${\text{AL}}_{\text{MET}}=\;{\text{A}}_{1}\cdot \;\tau_{1}$$ (3)

Where AL_MET_ is alactic anaerobic system estimated by the fast component of excess post-exercise oxygen consumption, $\dot{\text{V}}$O_2(t)_ is the oxygen uptake at time t, $\dot{\text{V}}$O_2baseline_ is the oxygen uptake at baseline, A is the amplitude, td is the time delay, *t* is the time constant, and _1_ and _2_ denote the fast and slow components, respectively.

### Statistical analysis

The distribution of physiological data was analyzed by Shapiro-Wilk’s test and the results showed a normal Gaussian distribution. Data were reported as means ± standard deviations. The effect of recovery times on AL_MET_ calculations was tested using one-way analysis of variance (ANOVA) with repeated measures. Comparison of the calculations obtained using the GEDAE-LaB and commercial computer program values was performed with paired *t*-test. Intra-investigator reliability was assessed using Intraclass Correlation Coefficient (ICC). Statistical analyses were performed using SPSS version 13.0. Statistical significance was set at *P* ≤ 0.05.

## Results

Physiological and mechanical parameters measured during maximal incremental exercise test are shown in Table 1. Power output measured at moderate and severe domains were 99.1 ± 13.6 W and 279.5 ± 40.6 W, respectively. All subjects completed the 10 min of exercise at moderate domain, while the time to exhaustion at severe domain was 144 ± 65 s. The mean of [La^-^]_peak_ measured during moderate and severe domains were 2.3 ± 0.3 mmol∙l^-1^ and 7.5 ± 2.2 mmol∙l^-1^, respectively.

**Table 1:** Measured parameters during maximal cycling incremental test (n = 11).

$\dot{\text{V}}$O_2_max (ml∙kg^-1^∙min^-1^)	39.4 ± 9.6
Maximal heart rate (bpm)	177 ± 15
Maximal power output (W)	250.0 ± 31.0
Ventilatory threshold (W)	125.1 ± 15.8
Ventilatory threshold (%$\dot{\text{V}}$O_2_max)	50.8 ± 8.5

Data are means ± standard deviations.

**Fig 1:**
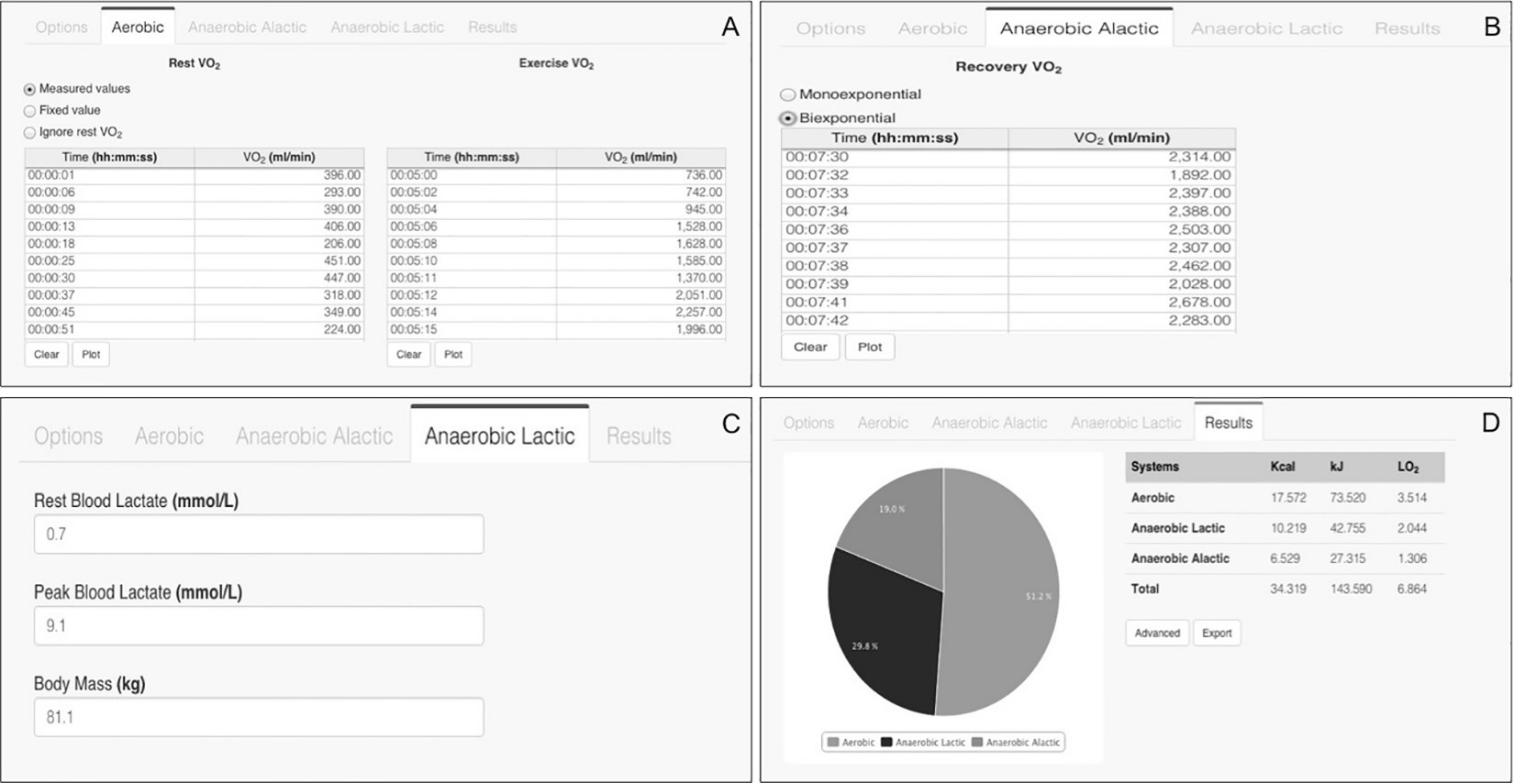
Templates of the GEDAE-LaB for input of physiological data. These templates are used to calculate aerobic (panel A), anaerobic alactic (panel B), and anaerobic lactic (panel C) systems contributions, as well as output data of energetic profile and energy expenditure amount (panel D) during severe domain.

Fig 1 shows templates of the GEDAE-LaB with a typical inputted physiological data and outcomes of energetic profiles and amount of energy expenditure using severe domain as an example. The results of the present study revealed that there were no statistical differences in the calculation of the AL_MET_ using different times of recovery for both moderate and severe domains (*P*> 0.05, Table 2). The Table 3 shows the calculation of the energy system contributions and total energy expenditure using GEDAE-LaB and commercial software. No statistical differences were found between the parameters obtained by these two software for both moderate and severe domains (*P* > 0.05). Tables 4 and 5 show intra-investigator variation for amount of energy expenditure and energy system contributions estimated at moderate and severe domains, respectively. The ICC revealed that these estimates were highly reliable for both domains (all ICC ≥ 0.95).

**Table 2:** Calculation of the anaerobic alactic system contribution during moderate and severe domains using different times of recovery.

	6 min	8 min	10 min
Moderate Domain			
Kcal	2.9 ± 0.5	2.9 ± 0.6	2.9 ± 0.6
kJ	12.2 ± 2.4	12.2 ± 2.8	12.2± 2.8
LO_2_	0.5 ± 0.1	0.5 ± 0.1	0.5 ± 0.1
Severe Domain			
Kcal	4.2 ± 2.4	3.3 ± 2.1	4.8 ± 2.5
kJ	17.8 ± 10.2	13.8 ± 9.1	18.9 ± 11.3
LO_2_	0.8 ± 0.4	0.6 ± 0.4	0.9 ± 0.5

Data are means ± standard deviations.

**Table 3:** Comparison of the energy system contributions and total energy expenditure determined by GEDAE-LaB and commercial software (n = 11).

	Moderate Domain		Severe Domain	
	GEDAE-LaB	Commercial Software	GEDAE-LaB	Commercial Software
**Aerobic**				
Kcal	53.2 ± 6.0	56.5 ± 8.5	23.3 ± 8.5	20.9 ± 9.1
kJ	222.8 ± 25.1	236.3 ± 35.6	97.8 ± 35.5	87.3 ± 38.1
LO_2_	10.6 ± 1.2	11.3 ± 1.7	4.6 ± 1.7	4.2 ± 1.8
%	93 ± 2	93 ± 2	65 ± 9	63 ± 12
**Anaerobic Lactic**				
Kcal	0.9 ± 1.0	0.9 ± 1.0	6.8 ± 2.3	6.8 ± 2.3
kJ	3.9 ± 4.4	3.9 ± 4.4	28.7 ± 9.9	28.7 ± 9.9
LO_2_	0.1 ± 0.2	0.1 ± 0.2	1.3 ± 0.4	1.3 ± 0.4
%	2 ± 2	2 ± 2	22 ± 8	22 ± 8
**Anaerobic Alactic**				
Kcal	2.9 ± 0.6	3.3 ± 0.7	4.8 ± 2.5	5.0 ± 2.8
kJ	12.2 ± 2.8	14.0 ± 2.8	18.9 ± 11.3	20.7 ± 11.5
LO_2_	0.5 ± 0.1	0.6 ± 0.1	0.9 ± 0.5	1.0 ± 0.6
%	5 ± 2	5 ± 2	13 ± 8	15 ± 8
**Total energy expenditure**				
Kcal	57.0 ± 2.5	60.7 ± 3.5	34.9 ± 4.4	32.7 ± 4.7
kJ	238.9 ± 10.7	253.9 ± 14.2	145.4 ± 18.9	136.7 ± 19.8
LO_2_	11.2 ± 0.5	12.0 ± 0.6	6.9 ± 0.9	6.5 ± 0.9

Data are means ± standard deviations.

**Table 4:** Intra-investigator variation for energy expenditure amount and energy system contribution estimated at moderate domain (n = 11).

	Investigator 1	Investigator 2	Investigator 3	Investigator 4	ICC
**Aerobic**					
Kcal	53.2 ± 6.0	53.2 ± 6.0	53.2 ± 6.0	53.2 ± 6.0	1.00
kJ	222.8 ± 25.1	222.8 ± 25.1	222.8 ± 25.1	222.8 ± 25.1	1.00
LO_2_	10.6 ± 1.2	10.6 ± 1.2	10.6 ± 1.2	10.6 ± 1.2	1.00
%	93 ± 2	93 ± 2	93 ± 2	93 ± 2	1.00
**Anaerobic Lactic**					
Kcal	0.9 ± 1.0	0.9 ± 1.0	0.9 ± 1.0	0.9 ± 1.0	1.00
kJ	3.9 ± 4.4	3.9 ± 4.4	3.8 ± 4.3	3.8 ± 4.3	1.00
LO_2_	0.1 ± 0.2	0.1 ± 0.2	0.1 ± 0.2	0.1 ± 0.2	1.00
%	2 ± 2	2 ± 2	2 ± 2	2 ± 2	1.00
**Anaerobic Alactic**					
Kcal	2.9 ± 0.6	2.9 ± 0.6	2.9 ± 0.6	2.9 ± 0.6	1.00
kJ	12.2 ± 2.8	12.2 ± 2.8	12.2 ± 2.8	12.2 ± 2.8	1.00
LO_2_	0.5 ± 0.1	0.5 ± 0.1	0.5 ± 0.1	0.5 ± 0.1	1.00
%	5 ± 2	5 ± 2	5 ± 2	5 ± 2	1.00
**Total energy expenditure**					
Kcal	57.0 ± 2.5	57.0 ± 2.5	57.0 ± 2.5	57.0 ± 2.5	1.00
kJ	238.9 ± 10.7	238.9 ± 10.7	238.9 ± 10.7	238.9 ± 10.7	1.00
LO_2_	11.2 ± 0.5	11.2 ± 0.5	11.2 ± 0.5	11.2 ± 0.5	1.00

Data are means ± standard deviations. ICC: Intraclass Correlation Coefficient.

**Table 5:** Intra-investigator variation for the amount of energy expenditure and energy system contribution estimated at severe domain (n = 11).

	Investigator 1	Investigator 2	Investigator 3	Investigator 4	ICC
**Aerobic**					
Kcal	23.3 ± 8.5	23.3 ± 8.5	23.3 ± 8.5	23.3 ± 8.5	1.00
kJ	97.8 ± 35.5	97.8 ± 35.5	97.8 ± 35.5	97.8 ± 35.5	1.00
LO_2_	4.6 ± 1.7	4.6 ± 1.7	4.6 ± 1.7	4.6 ± 1.7	1.00
%	65 ± 9	66 ± 9	68 ± 10	68 ± 10	0.97
**Anaerobic Lactic**					
Kcal	6.8 ± 2.3	6.8 ± 2.3	6.8 ± 2.3	6.8 ± 2.3	1.00
kJ	28.7 ± 9.9	28.7 ± 9.9	28.7 ± 9.9	28.7 ± 9.9	1.00
LO_2_	1.3 ± 0.4	1.3 ± 0.4	1.3 ± 0.4	1.3 ± 0.4	1.00
%	22 ± 8	22 ± 8	21 ± 8	21 ± 8	0.99
**Anaerobic Alactic**					
Kcal	4.8 ± 2.5	4.5 ± 2.6	3.8 ± 2.6	3.8 ± 2.6	0.95
kJ	18.9 ± 11.3	18.9 ± 11.3	16.1 ± 10.9	16.1 ± 10.9	0.95
LO_2_	0.9 ± 0.5	0.9 ± 0.5	0.8 ± 0.5	0.8 ± 0.5	0.95
%	13 ± 3	13 ± 8	11 ± 8	11 ± 8	0.94
**Total energy expenditure**					
Kcal	34.9 ± 4.4	34.6 ± 7.3	33.9 ± 4.5	33.9 ± 4.5	0.95
kJ	145.4 ± 18.9	145.4 ± 18.9	142.6 ± 18.7	142.6 ± 18.7	0.95
LO_2_	6.9 ± 0.9	6.8 ± 0.9	6.7 ± 0.9	6.7 ± 0.9	0.95

Data are means ± standard deviations. ICC: Intraclass Correlation Coefficient.

## Discussion

The estimate of energy expenditure is useful for the evaluation of acute and chronic effects of exercise in athletic performance and daily life. An understanding of energy metabolism involved in an athletic event or in daily activity is important for the correct structuring of strategies that target both health and athletic performances. However, the determination of energetic profiles using physiological data often require the use of some mathematical functions that may be considered unusual to many exercise physiologists. For example, previous studies have fitted post-exercise $\dot{\text{V}}{\text{O}}_{2}$ kinetics using a bi- or a mono-exponential model to determine the AL_MET_ during exercise [8,9,16]. This results in a more complex process to estimate the AL_MET_ energy expenditure because the fitting of the exponential data requires the use of appropriate equations and initial reference values for each parameter, which are not frequently provided by the commercial software. Considering that available software are not free and were not designed especially for the determination of energy expenditure, processing physiological data for establishment of energy profile during exercise might be considered costly, time-consuming, expertise-requiring and error-prone in their use. Therefore, the current study introduced GEDAE-LaB as software specifically designed for estimation of energetic profiles during dynamic exercise.

There are many methods proposed in literature to determine exercise-related energy expenditure, including questioners [25] and metabolic equivalent [23]. However, these indirect methods may be affected by some factors known to influence the energy expenditure, including age and gender [1]. In addition, these methods cannot distinguish contributions of aerobic and anaerobic systems to the total amount of energy expenditure. In turn, measurements of $\dot{\text{V}}{\text{O}}_{2}$ and lactate accumulation have been employed as one of the main methods to estimate energy system contribution during exercise [2,8,9]. This method has the additional advantage of enabling the establishment of energetic profiles in a large variety of non-laboratorial-based tests [13,14,15]. A recent review by Hall et al. [1] focused on technical aspects to provide data on activity-related energy expenditure and they concluded that literature fails to fully report energy expenditure in younger and older adults using adequate metric units in publications. These authors highlighted that the use of the metric units familiar to the scientific community (e.g. ml∙kg^-1^∙min^-1^ or Kcal) is fundamental to translate the findings of scientific studies to professional practices. Considering these aspects, GEDAE-LaB was developed to express both energy system contributions and energy expenditure results using the International System of Units (e.g. kJ), as well as metrics traditionally used in the exercise field (e.g. Kcal or liters of O_2_). Thus, it is possible that GEDAE-LaB serves a larger number of professionals by precisely calculating energy profile during exercise, expressing results using different metric units.

It is well recognized that during continuous exercises the ATP is resynthesized by aerobic and anaerobic systems in order to maintain a required power output [5,6,7]. The results of the present study demonstrated that A_MET_ accounted for 93 and 66% of total energy expenditure during moderate and severe domains, respectively. Consequently, anaerobic sources (AL_MET_ + LA_MET_) accounted for the remaining 7 and 34% for total energy expenditure, respectively. These estimates are in accordance with other investigations using the same method, but were performed in commercial software. For instance, Bertuzzi et al. [8] found approximately 67% A_MET_, 7% AL_MET_, and 26% LA_MET_ contributions in exercise performed at 110% of power output eliciting $\dot{\text{V}}$O_2_max in healthy male subjects (time to exhaustion ~155 s). In addition, no statistical difference was observed in energy system contributions and total energy expenditure as determined by GEDAE-LaB and commercial software. These findings suggest that GEDAE-LaB has an ability similar to existing software to calculate energy expenditure. It is important to note, however, that calculations of that study were performed using a software package not designed to estimate energy contribution, so different and complementary software packages were necessary (e.g. Excel) in order to conclude all analyses. It is also important to notice that previous studies have reported limitations regarding the use of [La^-^] and the fast component of excess post-exercise oxygen consumption to estimate anaerobic systems contribution. Gladden [26] indicated that O_2_ availability is only one of several interacting factors that cause the increase in blood lactate during exercise, whereas McMahon and Jenkins [27] suggested that more studies were necessary to elucidate the most dominant components governing the PCr resynthesis following muscular contraction. Nevertheless, it is important to recognize that the inexistence of a method universally accepted for measurement of anaerobic system during exercise, especially in non-laboratory tests, is still an unsolved problem.

In order to analyze a possible impact of the recovery time on AL_MET_ calculations, the AL_MET_ was estimated using the data of excess post-exercise oxygen consumption for 6, 8, and 10 min of the recovery. The results of the present study revealed there were no statistical differences in the calculation of the AL_MET_ using different times of recovery for both exercise domains (Table 2). These data suggest that to measure the $\dot{\text{V}}$O_2_ recovery during the first 6 min would be sufficient to determine the AL_MET_. This is in agreement with previous studies that suggested that the resynthesis of high-energy phosphate stores is completely restored during the first 2–3 min of recovery in both human [11] and animals [28]. It is believed the responses of the phosphocreatine and $\dot{\text{V}}$O_2_ during the recovery are temporally similar because the resynthesis of high-energy phosphate in the skeletal muscle is influenced by O_2_ availability [10, 29]. Thus, the users of the GEADE-LaB are encouraged to measure the $\dot{\text{V}}$O_2_ breath-by-breath, at least during the first 6 min of recovery to determine the AL_MET_.

GEDAE-LaB was intentionally designed in an easy-to-use concept to provide an intuitive interface between users and software. However, before using a new tool to calculate the energy expenditure, it is important to determine the intra-investigator variation. Intra-investigator reliability determines the degree of agreement among investigators and it is often statistically determined using ICC [30]. It has been suggested that when the ICC is used, values between 0.7–0.8 should be treated with caution, while values > 0.9 can be considered highly reliable [30]. In the current study, intra-investigator reliability for energy expenditure and energy system contribution calculated in both moderate and severe domains using GEDAE-LaB were highly reliable (all ICC ≥ 0.95). Considering that investigators with different experience levels used the GEDAE-LaB, these findings suggest that investigators had little difficulty in understanding the necessary procedures for the correct functioning of the software, regardless their familiarization with procedures.Therefore, GEDAE-LaB can be considered easy to use and understand by those minimally familiarized with procedures adopted for energy expenditure calculations.

It is important to acknowledge some limitations of the present study. First, it has been widely reported in literature that blood lactate concentration measured during exercise represents a balance between lactate production and clearance [3,4]. This suggests that the anaerobic lactic contribution determined by O_2_ equivalent from [La^-^] could be influenced by lactate clearance. In addition, the O_2_ equivalent from [La^-^] used in present study does not represent the exact stoichiometric relationship between lactate formation and ATP resynthesis. As a result, other O_2_ equivalents from [La^-^] (e.g. 5.3 ml O_2_∙kg^-1^∙mmol^-1^ lactate∙L^-1^) might also be used [31,32]. Therefore, the comparison of the anaerobic lactic contribution determined with different energy equivalents must be performed with caution. Second, technical support and detailed user manuals are not currently available in different languages for users of GEDAE-LaB. Third, physiological data must be inputted using English decimal separator, while duration time of $\dot{\text{V}}$O_2_ measurement during both exercise and recovery must be inputted as hh:mm:ss or seconds (s). Perhaps future users will find it necessary to format their personal computers in order for outputted data have this configuration. However, there are many other advantages of using GEDAE-LaB compared with existing software. GEDAE-LaB is available to all users free of charge and is intended for cross-platform use. Because it is available online, users have minimal system requirements to access the internet and, thus, GEDAE-LaB. This could help to remove some financial barriers of conducting research in the energy expenditure field. In addition, given that calculations may be directly performed by importing output data from metabolic systems into spreadsheets of GEDAE-LaB, it can be considered more efficient than existing software, because it eliminates the lengthy and costly steps of the data processing. GEDAE-LaB also offers the possibility of calculating the contributions of A_MET_, AL_MET_, and LA_MET_ individually or together. At least to our knowledge, this feature is not available in any other software package. Lastly, a future advantage is that such GEDAE-LaB is open-source software and in the future other mathematical functions traditionally used to determine physiological (i.e. maximal accumulated oxygen deficit) and mechanical (i.e. critical power concept) parameters related to energetics of exercise may be incorporated into the software.

In conclusion, considering that energy expenditure induced by exercise may guide interventions that promote health maintenance and athletic performance improvement, we introduced GEDAE-LaB as free and easy-operating software specifically developed to calculate energetic profile during exercise. Our findings suggest that GEDAE-LaB is an easily understood software for users minimally familiarized with procedures used for energy expenditure calculations. In addition, due to the open nature of the software, future studies could contribute for GEDAE-LaB expansion by the addition of new mathematical functions related to exercise physiology.
